# The accuracy of effect-size estimates under normals and contaminated normals in meta-analysis

**DOI:** 10.1016/j.heliyon.2019.e01838

**Published:** 2019-06-11

**Authors:** Philomena Marfo, G.A. Okyere

**Affiliations:** a African Institute for Mathematical Sciences (AIMS-Cameroon), Cameroon; b Kwame Nkrumah University of Science and Technology, Ghana

**Keywords:** Applied mathematics, Effects size, Meta-analysis, Accuracy, Statistical bias, Monte Carlo simulation, Experimental design, Statistics

## Abstract

This article evaluates the accuracy of effect-size estimates for some estimation procedures in meta-analysis. The dilemma of which effect-size estimate is suitable is still a problem in meta-analysis. Monte Carlo simulations were used to generate random variables from a normal distribution or contaminated normal distribution for primary studies. The primary studies were hypothesised to have equal variance under different population effect sizes. The primary studies were also hypothesised to have unequal variance. Meta-analysis was done on the simulated hypothesized-primary-studies. The effect sizes for the simulated design of the primary studies were estimated using Cohen's *d*, Hedges' *g*, Glass' △, Cliff's delta *d* and the Probability of Superiority. Their corresponding standard error and confidence interval were computed and a comparison of an efficient estimator was done using statistical bias, percentage error and confidence interval width. The statistical bias, percentage error and confidence interval width pointed to Probability of Superiority as an accurate effect size estimate under contaminated normal distribution, and Hedges' *g* as the most accurate effect size estimates compared to Cohen's *d* and Glass' △ when equal variance assumptions are violated. This study suggests that the accuracy of effect size estimates depends on the details of the primary studies included in the meta-analysis.

## 1 Introduction

Meta-analysis combines studies that answer the same underlying question using systematic and statistical methods to analyse and synthesise their results [1]. The most important part of meta-analysis is the effect size, which is the name given to a family of indices that measure the magnitude of treatment effect [2]. It is a measure that can inform judgement about the practical significance of a study.

Effect size estimates such as Cohen's *d*, Hedges' *g* and Glass △ depend on the assumption that studies are normally distributed and have equal variance [1], [3]. Previous studies have shown that these effects size tends to be biased when these assumptions are not met. In an attempt to investigate which of these effects size is better under the same conditions, several articles [4], [5], [6], [7] have explored this in different perspectives. The purpose of this study is to evaluate the performance of some effect size estimates using Monte Carlo simulations.

### 1.1 Estimated effect-size indices

Effect size is a simple way of quantifying the size of the difference between two groups. It allows us to move beyond the “Does it work?” question to “How well does it work in a range of contexts?”.

The effect-size indices Cohen's *d*, Hedge's *g*, and Glass' △ were used because they are easy to calculate and are popular. The robust effect sizes used were the Cliff's delta *d* and Probability of Superiority because they are also popular, although they are harder to calculate.

**Cohen's** *d***:**

This effect size is based on the standardised mean difference. We compute the Cohen's *d* for studies that used two independent groups. For each study made up of Group 1 and Group 2 in the data, we derive Cohen's *d* [1] as

(1) $$d=\frac{\overset{¯}{X_{1}}-\overset{¯}{X_{2}}}{S_{within}},$$

where $\overset{¯}{X_{1}}$ and $\overset{¯}{X_{2}}$ are the sample mean of the two groups, $S_{within}$ is the pooled standard deviation between the two groups, which is computed as

(2) $$S_{within}=\sqrt{\frac{(n_{1}-1)S_{1}^{2}+(n_{2}-1)S_{2}^{2}}{n_{1}+n_{2}-2}},$$

where $n_{1}$ and $n_{2}$ are the sample size of Group 1 and Group 2 respectively, and $S_{1}$, $S_{2}$ are the standard deviations of the Group 1 and Group 2 respectively [1].

The variance of Cohen's *d* ($V_{d}$) is given by

(3) $$V_{d}=\frac{n_{1}+n_{2}}{n_{1}n_{2}}+\frac{d^{2}}{2(n_{1}+n_{2})},$$

where *d* is the Cohen's *d* [1].

**Glass'** △**:**

This is an estimator of the effect size that uses only the standard deviation of the control group [8], defined by

(4) $$△=\frac{\overset{¯}{X_{1}}-\overset{¯}{X_{2}}}{S_{2}}$$

where $\overset{¯}{X_{1}},\overset{¯}{X_{2}}$ and $S_{2}$ are the mean of Group 1, the mean of Group 2 and the standard deviation of Group 2 [9].

Glass argued that if several treatments were compared to the control Group, it would be better to use just the standard deviation computed from the control Group so that effect sizes would not differ under equal means and different variances.

The variance of the Glass' △ effect size ($V_{△}$), which is also the same as the within-study variance, is given by

(5) $$V_{△}=\frac{n_{1}+n_{2}}{n_{1}n_{2}}+\frac{{△}^{2}}{2(n_{2}-1)},$$

where $n_{1}$ and $n_{2}$ are the sample sizes of Group 1 and Group 2, respectively [9].

**Hedges'** *g***:**

Hedges' *g* for the effect size using standard mean difference is given as

(6) g=J×d

where *J* is estimated as

(7) $$J=1-\frac{3}{4df-1},$$

with $df=n_{1}+n_{2}-2$, the degrees of freedom used in estimating $S_{within}$ and *d* is the Cohen's *d* calculated using Equation (1) [1].

The variance of the Hedge's *g* effect size ($V_{g}$), which is also the same as the within study variance, is given by

(8) $$V_{g}=J^{2}\times V_{d},$$

where $V_{d}$ is the variance of Cohen's *d* [1].

**Cliff's delta** *d***:**

Cliff's delta *d* computes the probability that a randomly selected observation from one Group is larger than a randomly selected observation from another Group, minus the reverse probability [10]. It is based on the ordinal properties of the data, and because it is not affected by rank, it preserves data transformation. The sample estimate of Cliff's delta *d* is estimated as:

(9) $$\text{Cliff's delta}\;d=\frac{\#(x_{i}>x_{j})-\#(x_{i}<x_{j})}{mn}$$

where $\#,x_{i},x_{j}$, are the number of times, Group 1 and Group 2 respectively and *m*, *n* are the number of observations in Group 2 and Group 1 respectively.

In Equation (9), the observation from each Group is compared to the other, and the number of times each observation is higher or lower than the other are counted; ties are not counted. The difference is then divided by the total number of comparisons.

A matrix can be obtained from the dataset, and a Cliff's delta *d* can be calculated as follows [11]: Let Group 1 and Group 2 be vectors in $R^{m\times n}$, such that Group 1 $\in R^{m}$, ∀m∈N and Group 2 $\in R^{n},\forall n\in N$. A matrix expressed by $\delta \in R^{m\times n}$ can be obtained by the function $\delta :(R^{m},R^{n})\to R^{m\times n}$ as illustrated in Equation (10)

(10) $$d_{ij}=\left\{ \begin{array}{cc} +1 & \to {\text{Group 1}}_{i}>{\text{Group 2}}_{j},\;\forall i,\forall j\\ -1 & \to {\text{Group 1}}_{i}<{\text{Group 2}}_{j},\;\forall i,\forall j\\ 0 & \to {\text{Group 1}}_{i}={\text{Group 2}}_{j},\;\forall i,\forall j. \end{array}\right.$$

Equation (10) generates a matrix of dimension m×n with only three possible values: +1,−1 and 0. The Cliff's delta *d* can be obtained by either computing means for each column or computing means for each row as follows:

(11) $$\text{Cliff's delta }d\text{ estimated with rows}=\frac{1}{mn}\sum_{m}^{i}\sum_{n}^{j}d_{ij}$$

or

(12) $$\text{Cliff's delta }d\text{ estimated with columns}=\frac{1}{nm}\sum_{n}^{j}\sum_{m}^{i}d_{ji}$$

where *m* and *n* are the number of observations for Group 2 and Group 1 respectively. The final value of this computing is the Cliff delta *d* as expressed in Equation (9).

Its variance is given by

(13) $$Var(\text{Cliff's delta}\;d_{i})=\frac{(m-1)S_{d_{i.}}^{2}+(n-1)S_{d_{.j}}^{2}+S_{d_{ij}}^{2}}{mn},$$

[10] where $d_{i.}$ is the marginal value of row *i*, $d_{.j}$ is the column marginal of column *j*, and $d_{ij}$ is the value of element *ij* in the matrix,

(14) $$d_{i\cdot }=\frac{\#(x_{i}>x_{j})-\#(x_{i}<x_{j})}{m},$$

(15) $$d_{\cdot j}=\frac{\#(x_{i}>x_{j})-\#(x_{i}<x_{j})}{n},$$

(16) $$S_{d_{i\cdot }}^{2}=\frac{\sum {\left(d_{i.}-\text{Cliff's delta}\;d\right)}^{2}}{m-1},$$

(17) $$S_{d_{\cdot j}}^{2}=\frac{\sum {\left(d_{.j}-\text{Cliff's delta}\;d\right)}^{2}}{n-1},$$

and

(18) $$S_{d_{ij}}^{2}=\frac{\sum \sum {\left(d_{ij}-\text{Cliff's delta}\;d\right)}^{2}}{\left(m-1)(n-1\right)}.$$

**Probability of superiority:**

This effect size measures the probability that a person picked at random from a treatment Group will have a higher score than a person picked at random from a control Group [3]. It is given parametrically as $Pr(X_{1}>X_{2})$ and it can be estimated as

(19) $$\overset{ˆ}{PS}=\frac{\overset{ˆ}{U}}{n_{1}n_{2}},$$

where $\overset{ˆ}{PS}$ is the estimated Probability of Superiority, *U* is the Mann–Whitney Statistic and $n_{1}$ and $n_{2}$ are the sample sizes.

The Variance of the Probability of Superiority is given as

(20) $$Var(\overset{ˆ}{PS})=\frac{1}{{\left(n_{1}n_{2}\right)}^{2}}Var(\overset{ˆ}{U}),$$

where $Var(\overset{ˆ}{U)}$ is the estimated variance of the Mann–Whitney U test when it is assumed that there are no ties. It is estimated as

(21) $$Var(\overset{ˆ}{U})=\frac{n_{1}n_{2}(n_{1}+n_{2}+1)}{12}.$$

### 1.2 Evaluation of accuracy of estimation procedures

The accuracy of estimation procedures was evaluated using statistical bias and percentage error.

**Statistical bias**

The term bias, in a statistical context, has a variety of meanings. These include selection bias, recall bias, estimation bias, systematic bias and observer bias. The one we are interested in is the estimation bias. Estimation bias is used to refer to the difference between the true or population value of a parameter being estimated from a sample and the sample value. A statistic is said to be unbiased if its mathematical expectation is the population parameter. The formula is

(22) Bias=E(Estimator)−population parameter.

**Percentage error**

Percentage error is the difference between the estimated value and the actual value when compared to the actual value expressed in per cent format. The formula is

(23) $$\text{Percentage error}=|\frac{\text{estimated value}-\text{actual value}}{\text{actual Value}}|\times 100.$$

A percentage very close to zero means the estimate is very close to the targeted value.

**Confidence interval width**

Confidence interval width is the distance from the upper limit to the lower limit of the confidence interval. It was estimated as

(24) CI width=Upper Limit of the CI−Lower Limit of the CI,

where CI is the confidence interval.

## 2 Methodology

For simplicity, Cohen's *d* is named as Cohen, Hedges' *g* as Hedges, Glass' △ as Glass, Cliff's delta *d* as Cliff and Probability of Superiority as PS in the tables, and the figures. The upper limit of the confidence interval is represented as U, while lower limit of the confidence interval is represented as L in the tables.

### 2.1 Design of the study

A total of five studies were considered for each condition investigated. The criteria for selecting the sample sizes was that the sum of the two sample sizes should be greater than the sum of the previous sample size. The sample sizes used for each of the five studies can be found in Table 1.

**Table 1:** Sample sizes for the five studies.

Study ID	n1	n2
1	10	8
2	15	20
3	30	18
4	40	50
5	120	80

For equal variance:

The parametric effect size (population effects size), *α* is defined as

(25) $$\alpha =\frac{\mu_{1}-\mu_{2}}{\sigma },$$

where $\mu_{1}$ is population mean of Group 1, $\mu_{2}$ is the population mean of Group 2 and *σ* is the standard deviation of either Group 1 or Group 2 [12].

For unequal variance:

(26) $$\alpha =\frac{\sigma_{2}\mu_{1}-\sigma_{1}\mu_{2}}{\sigma_{1}⁎\sigma_{2}},$$

where $\mu_{1}$ is population mean of Group 1, $\mu_{2}$ is the population mean of Group 2 and *σ* is the standard deviation of Group 1 and $\sigma_{2}$ is the standard deviation of Group 2.

Information used for the creation of each study can be found from Tables 2, 3, 4, 5, 6 and 7. ES is the population effect size.

**Table 2:** Properties of studies included for the Normal distribution.

ES	Both Normal Distr	Mean of Group 1	SD 1	Mean of Group 2	SD 2
0.1		50	5	49.5	5
0.2		60	5	59	5
0.5		80	6	77	6
0.8		80	7	74.4	7
1.20		45	3	41.4	3
2		120	10	100	10

**Table 3:** Properties of studies included for 1:2 unequal Variance.

ES	Unequal Variance	Mean of Group 1	SD 1	Mean of Group 2	SD 2
0.1	1:2	99.4	4	49.5	2
0.2		120.8	4	60	2
0.5		163	6	80	3
0.8		166.4	8	80	4
1.20		97.2	6	45	3
2		260	10	120	5

**Table 4:** Properties of studies included for 1:4 unequal Variance.

ES	Unequal Variance	Mean of Group 1	SD 1	Mean of Group 2	SD 2
0.1	1:4	198.8	8	49.5	2
0.2		241.6	8	60	2
0.5		326	12	80	3
0.8		332.8	16	80	4
1.20		194.4	12	45	3
2		520	20	120	5

**Table 5:** Properties of studies included for 1:5 unequal Variance.

ES	Unequal Variance	Mean of Group 1	SD 1	Mean of Group 2	SD 2
0.1	1:5	99.4	10	19.68	2
0.2		120.8	10	23.76	2
0.5		163	15	31.1	3
0.8		166.4	20	30.08	4
1.20		97.2	15	15.84	3
2		260	25	42	5

**Table 6:** Properties of studies included for 1:8 unequal Variance.

ES	Unequal Variance	Mean of Group 1	SD 1	Mean of Group 2	SD 2
0.1	1:8	99.4	16	12.224	2
0.2		120.8	16	14.7	2
0.5		163	24	18.875	3
0.8		166.4	32	17.6	4
1.20		97.2	24	8.55	3
2		260	40	22.5	5

**Table 7:** Properties of studies included of 1:10 unequal Variance.

ES	Unequal Variance	Mean of Group 1	SD 1	Mean of Group 2	SD 2
0.1	1:10	99.6	20	9.76	2
0.2		120.8	20	11.68	2
0.5		163	30	14.8	3
0.8		166.4	40	13.44	4
1.20		197.2	30	13.72	3
2		260	50	16	5

### 2.2 Information on the primary studies

All the variables in Table 2 satisfy Equation (25). This table shows the population mean and standard deviation of the two groups in each study with their respective population effect size. ES is Effect Size.

All the variables in Table 3 satisfy Equation (26). This table shows the population mean and standard deviation of the two groups in each study with their respective population effect size.

All the variables in Table 4 satisfy Equation (26). This table shows the population mean and standard deviation of the two groups in each study with their respective population effect size.

All the variables in Table 5 satisfy Equation (26).

All the variables in Table 6 satisfy Equation (26).

All the variables in Table 7 satisfy Equation (26).

### 2.3 Simulation

We used Monte Carlo simulations to simulate primary studies under known population conditions and then combined in a meta-analysis. Monte Carlo simulations were used so that it will be easier to manipulate the study design [13]. We considered the following factors.

**The pre-specified effect size:**

We used “cohd2delta”, which is estimated as

return(((2 * pnorm(d/2)) - 1)/pnorm(d/2))

in the “orddom” package in R to find the equivalence of Cohen's *d* in Cliff's delta *d* and “delta2cohd”, which is also estimated as

return(qnorm(-1/(d - 2)) * 2)

to find the equivalence of Cliff's delta *d* in Cohen's *d*. It also used the code

**Figure:**
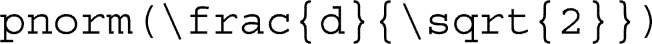

in R to find the equivalence of Cohen's *d* in Probability of Superiority [14] and the code

qnorm(PS)*sqrt(2)

to find the equivalence of PS in Cohen's *d*, where *d* is the population's Cohen's *d* and pnorm is the cumulative distribution function of the standard normal distribution. We used the following pre-specified population effects size.

1. α=0.10: ≡0.10 Cohen's *d* ≡0.0767 Cliff's delta d≡0.53 PS
2. α=0.2: ≡0.2 Cohen's *d* ≡0.1476 Cliff's delta d≡0.56 PS
3. α=0.50: ≡0.50 Cohen's *d* ≡0.3297 Cliff's delta d≡0.64 PS
4. α=0.8: ≡0.80 Cohen's *d* ≡0.4742 Cliff's delta d≡0.71 PS
5. α=1.20: ≡1.20 Cohen's *d* ≡0.6221 Cliff's delta d≡0.80 PS
6. α=2.0: ≡2.0 Cohen's *d* ≡0.8114 Cliff's delta d≡0.92 PS,

corresponding to Cohen's *d* suggestion and the expansion of Sawilowsky (2009) as very small, small, medium, high and very high respectively [15], [16].

**Population distribution**

We considered the situations where both groups are normally distributed, 5% contaminated normal distribution of each group, 10% contaminated normal distribution of each group and 15% contaminated normal distribution of each group.

**The variance ratios,** $\sigma_{2}:\sigma_{1}$

We used a variance ratio of 1:1, 1:2, 1:4, 1:5, 1:8 and 1:10.

**Figure 1:**
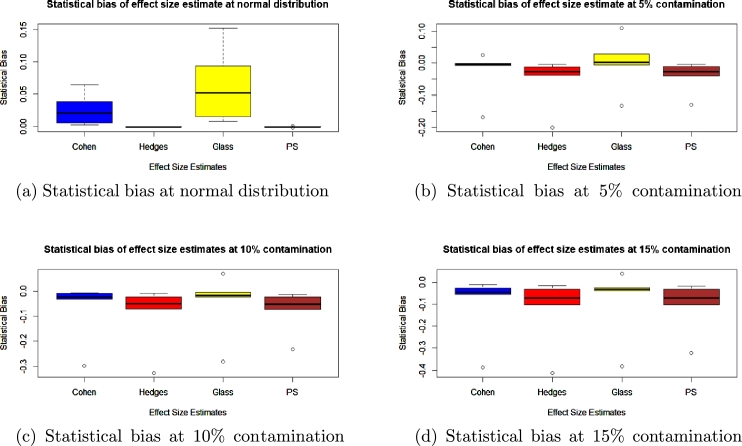
Statistical bias of effect Sizes at normal and different levels of contamination.

**Figure 2:**
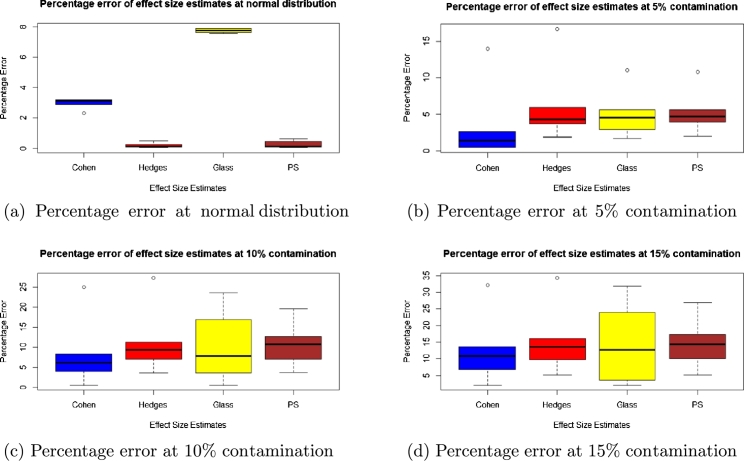
Percentage error of effect sizes at normal distribution and different levels of contamination.

**Figure 3:**
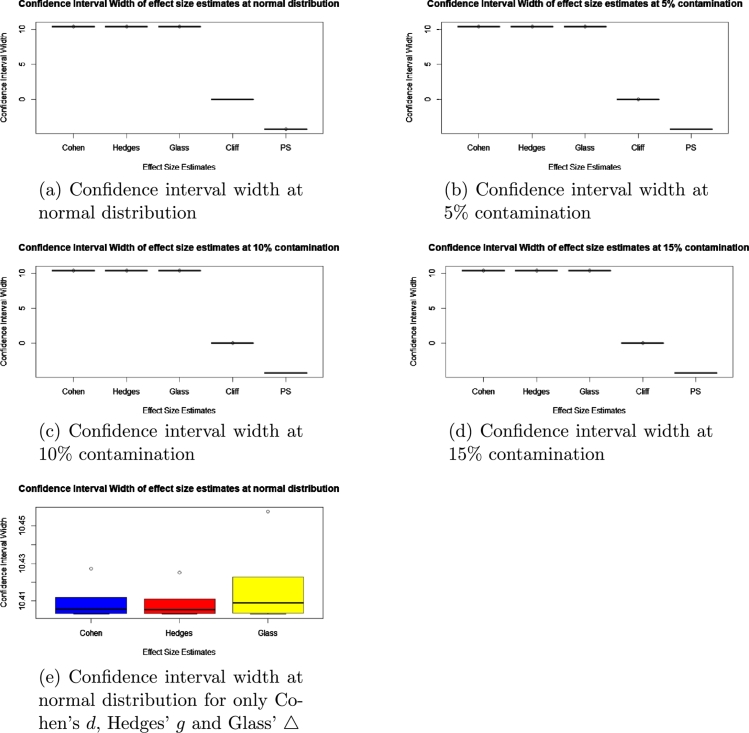
Confidence interval width of effect sizes at normal and different levels of contamination.

**Figure 4:**
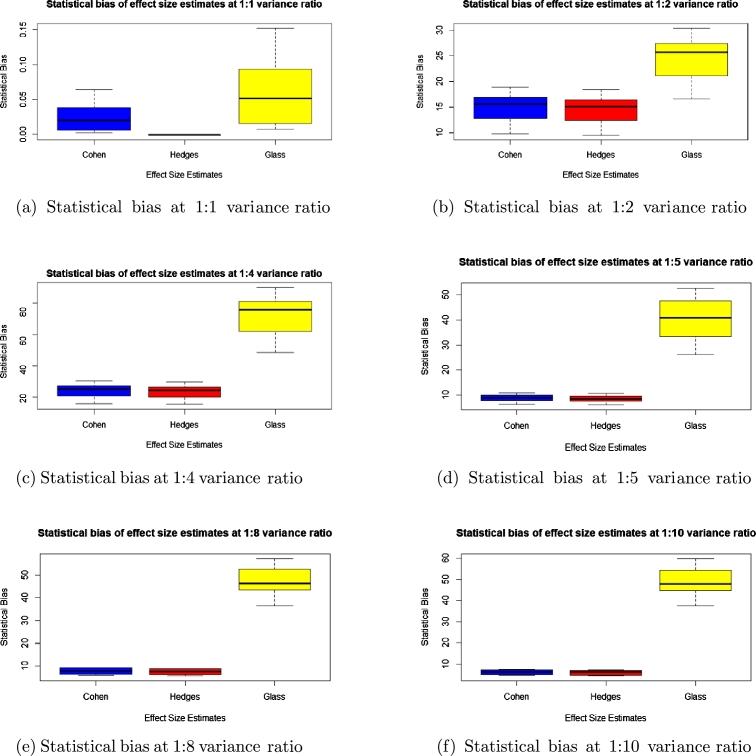
Statistical bias of effect sizes estimates at unequal variance ratio.

**Figure 5:**
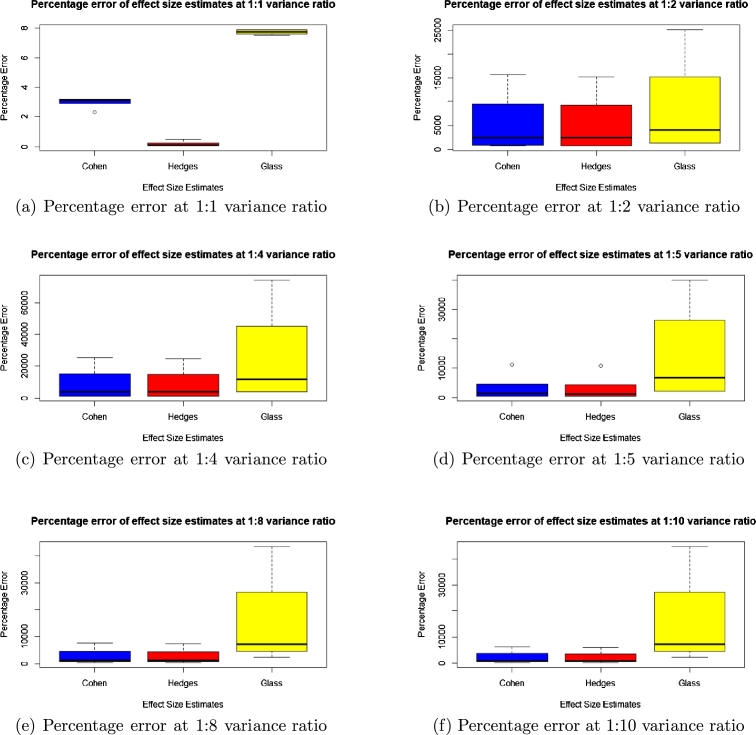
Percentage error of effect sizes at unequal variance ratio.

**Figure 6:**
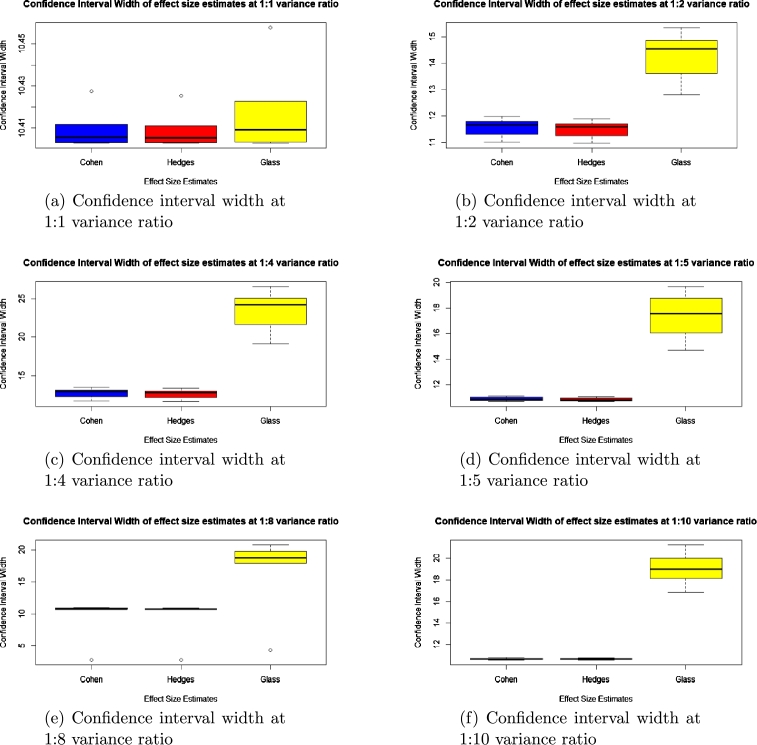
Confidence interval width of effect sizes at unequal variance.

To check for accuracy, we evaluated the statistical bias, percentage error, confidence interval width and standard error for each of the methods and presented the results in the boxplots in Figs. 1, 2, 3, 4, 5 and 6.

The guidance for the selection of the design factors and their values investigated in this simulation study was based on the work done by Harwell [17].

For each of the simulated meta-analysis, the population mean effect size was estimated using Cohen's *d*, Hedges' *g*, Glass' △, Cliff's delta *d* and PS. Each of the effects sizes were combined as

(27) $$\overset{¯}{Y}=\frac{\sum w_{i}\overset{ˆ}{Y_{i}}}{\sum w_{i}},$$

where $w_{i}$ is the weight of study *i* and is given as the inverse of the estimated sampling variance of any of the effect size obtained in the $i^{th}$ study:

(28) $$w_{i}=\frac{1}{\overset{ˆ}{v_{i}}}.$$

We used the inverse variance weighting method, where $v_{i}$ is the within-study error (sampling error), which is the same as the variance of any of the methods of effects size estimates discussed in Section 1.1. In addition, we also estimated the confidence interval for each method using $\overset{ˆ}{Y_{i}}\pm Z_{\alpha /2}\sigma_{\overset{ˆ}{Y}}$, where $\sigma_{\overset{ˆ}{Y}}$ is the estimated variance of the mean effect size and the *Z* value is the appropriate critical value from the unit normal distribution.

Normally distributed random variables were generated using the rnorm random number generator in R. We used the same seed value for the random number generator for each execution of the program with different sample size.

For the contamination of the normal distribution, we used a function that took six arguments to generate contaminated random samples: it first generates two random variables (z0 and z1) from the normal distributions. Binomial random variables (flag) were also generated, from which the two normally distributed random variables were combined using

z <- zo*(1-flag) + z1*flag,

where $z_{0}$ and $z_{1}$ are two normally distributed random variables generated and flag is the binomial random variable.

### 2.4 An example

This illustrative example was done with a code on Github https://github.com/philomena-aims/R-code-for-meta-analysis/blob/master/Individual_Studies.R and results achieved by changing the sample sizes n1 and n2 for each run. The value for Cliff's delta *d* and the PS are all converted to Cohen's *d*. The value in the bracket at the PS column is converted to the Probability of Superiority.

**Estimates of the mean effect size of the normal distribution studies with equal variance**

Using Equation (25) with ES=0.1, $\mu_{1}=mx=50$ and σ=sdx=sdy=5 $\mu_{2}=my=49.5$ was calculated. The sample sizes n1 and n2 were changed for each run.

Table 8 presents the estimated mean effect size of studies from the normal distribution with equal variance and a population effect size of 0.1. We can see that all the effect-size estimators were able to compute the effect size that is close to the population effect size except Cliff's delta *d* when the sample sizes were 10 and 8.

**Table 8:** Estimated mean effect size of studies from the normal distribution and equal variance by population effect size (0.1) and sample sizes.

Study ID	n1	n2	Cohen	Hedges	Glass	Cliff	PS
1	10	8	0.102	0.098	0.111	0.303	0.098 (0.527)
2	15	20	0.099	0.097	0.101	0.080	0.097 (0.527)
3	30	18	0.106	0.104	0.108	0.083	0.104 (0.529)
4	40	50	0.101	0.100	0.101	0.075	0.100 (0.528)
5	120	80	0.099	0.099	0.100	0.073	0.099 (0.528)

Table 9 presents the corresponding standard deviation of studies from the normal distribution with equal variance and a population effect size of 0.1 of the estimated mean effect size. We can see that the standard deviation was small for the PS and Cliff's delta *d* and was similar for Cohen's *d*, Hedges' *g* and Glass' △.

**Table 9:** Estimated standard deviation of studies from the normal distribution and equal variance with the population effect size (0.1) of studies and sample size.

Study ID	n1	n2	Cohen	Hedges	Glass	Cliff	PS
1	10	8	0.038	0.03796	0.037978	0.003167	−4.2248(0.00149)
2	15	20	0.068	0.06831	0.068316	0.002491	−4.3702(0.001)
3	30	18	0.054	0.05367	0.053669	0.002167	−4.4286(0.0008)
4	40	50	0.106	0.10607	0.106066	0.001540	−4.5700(0.0006)
5	120	80	0.115	0.11547	0.115470	0.001045	−4.7246(0.000417)

Table 10 presents the corresponding confidence interval of studies from the normal distribution with equal variance and a population effect size of 0.1 of the estimated mean effect size. We can see that the confidence interval was small for the PS and Cliff's delta *d* and was similar for Cohen's *d*, Hedges' *g* and Glass' △.

**Table 10:** Estimated confidence interval of studies from the normal distribution and equal variance with the population effect size (0.1) of studies and sample size.

	n1	n2	CohenL	CohenU	HedgesL	HedgesU	GlassL	GlassU	CliffL	CliffU	PSL	PSU
1	10	8	0.028	0.177	0.023	0.172	0.036	0.185	0.295	0.311	0.088 (0.5247)	0.107 (0.530)
2	15	20	-0.034	0.233	-0.037	0.231	-0.033	0.235	0.075	0.085	0.090 (0.525)	0.104 (0.529)
3	30	18	0.001	0.211	-0.001	0.209	0.003	0.213	0.078	0.087	0.098 (0.528)	0.110 (0.531)
4	40	50	-0.107	0.309	-0.108	0.308	-0.107	0.309	0.072	0.079	0.095 (0.527)	0.104 (0.529)
5	120	80	-0.127	0.326	-0.127	0.325	-0.126	0.326	0.071	0.075	0.096 (0.5269)	0.101 (0.5285)

Each of these effect sizes was combined in a meta-analysis with 95% confidence interval, and the accuracy of the estimated effects size was evaluated in terms of the statistical bias, and percentage error for the point estimates and confidence interval width for the interval estimates. The code for estimation of the accuracy of effect size estimates is on Github https://github.com/philomena-aims/R-code-for-meta-analysis/blob/master/Metanalysis.R.

## 3 Results

Comparing the effects sizes by the statistical bias, percentage error and confidence interval width at the various conditions investigated to examine the accuracy of each of the effect size methods used. The results are presented in Figs. 1, 2, 3, 4, 5 and 6.

In all the results, Cliff's delta *d* shows more variability and higher median in the boxplots, which was likely due to the smaller sample size we choose for the first study (n1+n2<30). Hence we decided to take Cliff's delta *d* out of the boxplot in order to see the information from the other effect size more clearly.

### 3.1 Results for normal distribution and different level of contamination

#### 3.1.1 Statistical bias

Looking at Fig. 1, at Normal distribution and no contamination, the statistical bias of Hedges' *g* and Probability of Superiority has the median closest to zero, followed by Cohen's *d* and lastly Glass' △. The interquartile range is different for the effect sizes (as shown by the length of the boxes), as Glass' △ shows more variability, followed by Cohen's *d*, then Hedges' *g* and lastly PS. The overall range of statistical bias (as indicated by the ends of the Whiskers for each boxplot) also displays similar behaviour to the interquartile range.

At different percentage of contamination, at 10% and 15% contamination respectively, the effect size with the median of its statistical bias closest to zero is Glass' △, followed by Cohen's *d*, then Hedges' *g* and finally PS. Hedges' *g* and PS have similar interquartile ranges, but Glass' △ has the smallest interquartile range. All the boxplots of the effect sizes show the presence of outliers in them, but Glass' △ has two outliers with one above all the boxplots and one outlier below. Cohen's *d*, PS and Hedges' *g* have an outlier which is below their boxplots. The outlier of PS is closest to its boxplot followed by Glass' △, then Cohen's *d* and lastly Hedges' *g*.

In Fig. 1b, 5% contamination, the effect size with the median of its statistical bias closest to zero is Glass' △, followed by Cohen's *d* then Hedges' *g* and lastly PS just as in the 10% and 15% contamination cases. But Hedges' *g*, Glass' △ and PS have similar variability in the middle 50% in the boxplots of their statistical bias whiles Cohen's *d* has smaller variability in the middle 50% of its statistical bias compared to the others. The overall boxplot(as shown by the ends of the whiskers) also displays the same behaviour as the interquartile range.

It was also observed that, as the percentage of contamination increases, the interquartile range of the statistical bias increases for Cohen, Hedges' *g* and PS but decreases for Glass' △. This decrease for Glass' △ is because the denominator in the formula of Glass' △ uses only the standard deviation of the control and not the pooled standard deviation as with Cohen's *d* and Hedges' *g*.

#### 3.1.2 Percentage error

In Fig. 2, at normal distribution, the effect size with the lowest percentage error is Hedges' *g* followed by PS then Cohen's *d* and lastly Glass' △. At the different percentages, the effect size with the lowest percentage error was the PS followed by Glass' △ then Cohen's *d* and lastly Hedges' *g* at 5% contamination but for 10% and 15% contamination it was PS followed by Cohen's *d* then Hedges' *g* and lastly Glass' △.

We also observed that, as the percentage of contamination increases, the variability in the percentage error of effects size increases in the context of this research.

#### 3.1.3 Confidence interval width

Fig. 3 shows that the effect size with the smallest confidence interval width under different contamination is the PS, followed by Cliff's delta *d* and lastly by the Glass' △, Hedges' *g* and Cohen's *d*. To check the differences between the confidence interval width clearly, we plotted the confidence interval width leaving out Cliff's delta *d* and PS in Fig. 3e. Fig. 3e shows that Hedges' *g* had the lowest confidence interval, followed by Cohen's *d* and lastly Glass' △.

### 3.2 Results for unequal variance ratios

Because of conversion constraints, we compared only three effect size estimates, namely Cohen's *d*, Hedges' *g* and Glass' △ for this section.

#### 3.2.1 Statistical bias of unequal variance

Looking at Fig. 4, the effect size with the statistical bias closest to zero in all the different variance ratio is Hedges' *g* followed by Cohen's *d* and lastly Glass' △.

#### 3.2.2 Percentage error for unequal variance

Looking at Fig. 5, the effect size with the lowest percentage error in all the different variance ratio is Hedges' *g* followed by Cohen's *d* and lastly Glass' △.

#### 3.2.3 Confidence interval width for unequal variance

From Fig. 6, Hedges' *g* has the smallest confidence interval width in comparison to Cohen's *d* and Glass' △ in the context of this article.

## 4 Discussion

The literature tells us that effect-size estimates turn out to be biased when assumptions of equal variance and normality are violated but is silent on how each one of these effect sizes behaves side by side when these assumptions are violated. Investigating the accuracy of these effect sizes may help researchers decide on which of these effect size to use with its known consequences under such circumstance. In this study, Monte Carlo simulations were used to simulate studies in R under known conditions and were manipulated by different variance ratios and different levels of contamination of the normal distribution. Through the comparison of the boxplots of statistical bias, percentage error, confidence interval width and standard error. We identified the following.

Hedges' *g* is an unbiased effect size of Cohen's *d* [1], and therefore we expect it to be a better estimator than Cohen's *d* and Glass' △ under no contamination and equal variance, which is consistent with our findings. Probability of Superiority and Cliff's delta *d* are known to be non-parametric effect-size estimates [3], which are therefore expected not to be affected by different levels of contamination and equal variance, and this is also consistent with our report with Probability of Superiority and Cliff's delta *d*.

Cliff's delta *d* did not perform very well in the context of this research because of the sample size used for our studies. We could see that when sample sizes were small (n1+n2<30), Cliff's delta *d* could not estimate the effect size close to the pre-specified population effect size.

Limitations of this study are that the number of studies is small (5) and we did not check the effect of the sample sizes on the effect sizes used in the design of the simulation. Future studies could include the number of studies and the choice of sample sizes in the factors, since it could affect the performance of each of these effect sizes.

## 5 Conclusion

Based on the objective of this article, the Probability of Superiority was the most accurate effect–size estimate under normal distribution and equal variance. It was also the most accurate under contaminated normals and equal variance. Furthermore, Cohen's *d d*, Hedges' *g*' *g* and Glass' △ performed very well in terms of accuracy under normal distribution and equal variance. They also performed very well in terms of accuracy under contaminated normals and equal variance even though they were not the most accurate.

This study concludes that, in the presence of different levels of contamination, Probability of Superiority is the most accurate estimate. However, the estimates of Cliff's delta *d* and Probability of Superiority are not usable in practice for a meta-analysis, as the information needed to compute them (and their variance) is essentially never reported. Cohen's *d* could be used, since it is the next most accurate. Also, at different levels of variance ratio, Hedges' *g* was the best estimate to use compared to Cohen's *d* and Glass' △.

Under normal and unequal variance, we examined only the accuracy of Cohen's *d*, Hedges' *g* and Glass' △. Hedges' *g* was the most accurate of the effect size estimates based on the statistical bias, percentage error, confidence interval width and standard error.

It was also observed in the context of this research that, at unequal variance ratios, Hedges' *g*, Cohen's *d* and Glass' △ estimated effect size that was too high or too low from the prespecified population effect size. This higher estimation of the effect sizes was clear in the column of the y–axis in the graph of the statistical bias and percentage error, which is consistent with what the literature says about these effect sizes being biased when these assumptions are violated.

This research suggests that the accuracy of effect-size estimates depends on the details reported in primary studies.

## Declarations

### Author contribution statement

Philomena Marfo: Conceived and designed the experiments; Performed the experiments; Analyzed and interpreted the data; Wrote the paper.

Gabriel A. Okyere: Contributed reagents, materials, analysis tools or data.

### Funding statement

This work was supported by Post AIMS bursary (grant number AIMS-Ghana 15/16.

### Competing interest statement

The authors declare no conflict of interest.

### Additional information

No additional information is available for this paper.
